# Sodium butyrate supresses malignant human mast cell proliferation, downregulates expression of KIT and promotes differentiation

**DOI:** 10.3389/falgy.2023.1109717

**Published:** 2023-03-10

**Authors:** Clayton A. MacDonald, Hui Qian, Priyanka Pundir, Marianna Kulka

**Affiliations:** ^1^ Department of Laboratory Medicine and Genetics, Trillium Health Partners, Mississauga, ON, Canada; ^2^ Nanotechnology Research Centre, National Research Council Canada, Edmonton, AB, Canada; ^3^ Department of Molecular and Cellular Biology, College of Biological Science, University of Guelph, Guelph, ON, Canada; ^4^ Department of Medical Microbiology and Immunology, Faculty of Medicine, University of Alberta, Edmonton, AB, Canada

**Keywords:** KIT, histone deacetylase inhibitors, cell cycle, proliferation, viability

## Abstract

Sodium butyrate (NaBu) is a class I histone deacetylase inhibitor (HDACi) that can impede the proliferation of transformed cells. Although some HDACi downregulate the expression of the stem cell factor receptor (KIT/CD117), the effect of NaBu on KIT expression and human mast cell proliferation requires further elucidation. In this study, we examined the effects of NaBu on three transformed human mast cell lines, HMC-1.1, HMC-1.2 and LAD2. NaBu (100 µM) inhibited the proliferation and metabolic activity of all three cell lines without significantly affecting their viability, suggesting that although the cells had ceased to divide, they were not yet undergoing apoptosis. Cell cycle analysis using the cell-permeant dye, propidium iodide, indicated that NaBu significantly blocked the cell cycle progression of HMC-1.1 and HMC-1.2 from G1 to G2/M phases. Furthermore, NaBu downregulated the expression of *C-KIT* mRNA and KIT protein expression in all three cell lines, but this effect was most significant in the HMC-1.1 and HMC-1.2, both of which harbour activating mutations in *KIT*, which proliferate more rapidly than LAD2. These data support earlier observations showing that human mast cell lines are sensitive to histone deacetylase inhibition. However, our data presents the novel observation that inhibition of cell proliferation by NaBu was not associated with a loss in cell viability but rather an arrest of the cell cycle. Higher concentrations of NaBu led to modest increases in histamine content, tryptase expression, and granularity. In conclusion, NaBu treatment of human mast cell lines led to a modest enhancement of the hallmarks of mature mast cells.

## Introduction

Sodium butyrate (NaBu), a short-chain fatty acid produced by the bacterial fermentation of dietary fibre in the intestine, has been reported to exert anti-neoplastic effects in many tumors, including colorectal cancer, breast cancer, and prostate cancer. NaBu is a histone deacetylase (HDAC) inhibitor and modulates the expression of 7%–10% of the genes in human cancer cells (1). HDACs, also called lysine deacetylases (KDAC), catalyze the removal of acetyl groups from *ε*-N-acetyl lysine amino acid on a histone or non-histone proteins, causing changes in chromatic structure and thus gene expression (2). HDACs modulate the expression of multiple proteins involved in cancer initiation and progression and may play a role in resistance to chemotherapy (3, 4). Mutation and/or aberrant expression of HDACs is often observed in numerous human cancers, making them anti-cancer therapeutic targets (4). HDAC inhibitors (HDACi) are a class of drugs that cause phenotypic changes in transformed cells (5), including growth arrest by disrupting the expression of numerous genes (1), yet most of the currently approved HDACi are wide-spectrum with poor clinical outcomes and numerous side-effects (6).

Unlike most HDACi, NaBu can be well-tolerated (7) and can be generated in the gut by microbes (8), maintaining gut health and protecting the host from disease (9–11). Similar to other HDACi, NaBu inhibits the proliferation of transformed cells by reducing the expression of cyclins, key regulators of the cell cycle (12), and arresting cells in the G0/G1 phase (13). Some very recent studies show that butyrate can also regulate miroRNA (miRNA) expression, and miRNA target genes that ultimately cause enhanced apoptosis, decreased proliferation, and promote cell-cycle arrest of colorectal cancer cells (14). For this reason, NaBu has been suggested as a combinational therapy to treat colon and other cancers (15–18).

The transformed cell line human mast cell-1 (HMC-1), derived from a patient with mast cell leukaemia, was the first established human mast cell line exhibiting a phenotype similar to that of human mast cells (19). HMC-1 cells express the *γ*-chain of the high affinity immunoglobulin E (IgE) receptor (Fc*ε*RI), but not the *α* and *β*-chains of Fc*ε*RI (20). HMC-1 cells express several mast cell-related markers such as the serine protease, *β*-tryptase (21), as well as heparin, chondroitin sulphate, and KIT (CD117), the receptor for stem cell factor (SCF) (19). Two point mutations in codons 560 and 816 of one allele of the proto-oncogene *C-KIT* causes constitutive tyrosine phosphorylation and activation of the KIT receptor (22, 23) allowing HMC-1 cells to proliferate independent of SCF. In most patients with systemic mastocytosis (SM), including aggressive SM (ASM) and mast cell leukemia (MCL), neoplastic cells express the oncogenic *C-KIT* mutation D816V (24), which confers resistance to imatinib (25, 26). Cladribine (2CdA) is a nucleoside analog that has been introduced as a promising agent for treatment of advanced SM but it does not counteract the kinase activity of *C-KIT* D816V or downstream signaling molecules (27). Therefore, a compound that alters neoplastic mast cell proliferation by targeting *C-KIT* expression or downstream KIT signalling, offers insight into new therapeutic approaches for mastocytosis.

The cell line Laboratory of Allergic Disease 2 (LAD2) is a more recently isolated mast cell line originating from the bone marrow of a patient with mastocytoma (28). Compared to HMC-1, this cell line resembles a more mature mast cell phenotype and expresses a functional Fc*ε*R1 receptor in which can stimulate degranulation upon cross-linking by IgE/antigen complexes. LAD2 also express KIT but, unlike HMC-1, LAD2 cells require SCF for survival and proliferation.

Previous studies have shown that other HDACi impaired proliferation and induced apoptosis in a cell cycle-dependent manner in a series of canine mast cell lines (29, 30) and HMC-1 cells (31). A novel HDACi, AR-42, downregulated expression of KIT mRNA and signaling through the KIT pathway, ultimately resulting in apoptosis of mouse P815 and canine C2 cells (29). Butyrate inhibited the proliferation of a mouse mastocytoma P815 by causing cell cycle arrest and apoptosis, similar to the HDACi trichostatin A, and reduced signaling through the KIT receptor by decreasing its expression (32). The precise effect of NaBu on HMC-1 or LAD2 human mast cell proliferation and KIT expression has not been previously examined. An older study found no effect of butyric acid on *C-KIT* mRNA expression by HMC-1 cells (33).

Given that NaBu impairs the proliferation of transformed cells, we hypothesized that NaBu would modify HMC-1 cell proliferation by affecting the expression of KIT and that this effect might be influenced by mutations in *C-KIT.* Herein, we examined the effect of NaBu treatment on three human mast cell lines: (1) imatinib-sensitive HMC-1.1 cells harboring the V560G mutation in the juxtamembrane domain of KIT, (2) imatinib-resistant HMC-1.2 cells harboring both the V560G and D816V mutations, and (3) LAD2 cells which do not possess either of these mutation in KIT. Our data demonstrates that NaBu blocked HMC-1.1, HMC-1.2 and LAD2 cell proliferation with an accompanying downregulating of KIT expression while causing cell cycle arrest. HMC-1.1 were the least sensitive and LAD2 were the most sensitive to NaBu effects, suggesting that mutations in *C-KIT* may influence NaBu-dependent epigenetic modifications.

## Materials and methods

### Cell culture

HMC-1.1 and HMC-1.2 human mast cell lines were cultured in Iscove's Modified Dulbecco's Medium (Lonza, Mississauga, ON, Canada) containing 10% FBS, 100 U/ml penicillin and 100 µg/ml streptomycin. Cells were maintained at 1  ×  10^5^ cells/ml at 37°C and 5% CO_2_. The LAD2 cell line was cultured in StemPro-34 SFM media (Life Technologies, Burlington, ON, Canada) supplemented with 2 mM L-glutamine, 100 U/ml penicillin, 50 mg/ml streptomycin, and 100 ng/ml recombinant human SCF (Peprotech, Rocky Hill, NJ). Cells were maintained at 1  ×  10^5^ cells/ml at 37°C and 5% CO_2_ and periodically tested for expression of KIT and Fc*ε*RI by flow cytometry. The cells were used within 8 weeks of thawing from cryopreservation.

### Trypan blue cell viability

HMC-1.1, HMC-1.2 and LAD2 cells were seeded at 1.5  ×  10^4^ cells/ml, 2  ×  10^4^ cells/ml and 5  ×  10^4^ cells/ml respectively in fresh media, treated with 100 µM NaBu (Sigma-Aldrich, Oakville, ON, Canada) and counted every 2 days with a hemocytometer using trypan blue exclusion dye. Cells counts and viability were plotted.

### Cell metabolic activity (XTT) assay

HMC-1.1, HMC-1.2 and LAD2 cells were seeded at 5  ×  10^4^ cells/well in a 96-well plate. Following treatment with NaBu (100 µM unless otherwise indicated), 50 µl of (2,3-bis-(2-methoxy-4-nitro-5-sulfophenyl)-2H-tetrazolium-5-carboxanilide) or XTT reagent (Roche, Laval, QC, Canada) was added to each well and incubated for 4 h at 37°C and 5% CO_2_. Absorbance at A_405_ was measured (Reference A_690_) on a Varioskan plate reader (ThermoFisher Scientific, Mississauga, ON, Canada) and expressed as percent of control (untreated cells).

### Cell cycle assay

HMC-1.1, HMC-1.2, and LAD2 cells were suspended at 5  ×  10^5^ cells/ml in fresh media and treated with NaBu (100 µM). Subsequently, 2  ×  10^5^ cells were fixed in ice cold 70% ethanol for 2 h then resuspended in PBS. Cells were then permeabilized, treated with RNase and stained for 30 min with propidium iodide (Sigma-Aldrich) in 0.1% Triton X-100. Fluorescence of stained cells was analyzed on a FACSArray (BD Biosciences, Mississauga, ON, Canada) and cell cycle analyzed using FlowJo software (Tree Star, Ashland, OR). Results are presented as percent of cells in G2/M phase of the cell cycle.

### Quantitative PCR analysis

HMC-1.1, HMC-1.2 and LAD2 were treated with NaBu (0–1000 µM) for 3 days at 37°C and 5% CO_2_. Total RNA was isolated from each treatment using Trizol Reagent (Sigma-Aldrich) based on manufacturer's protocol and 1 µg of total cellular RNA was reverse transcribed into cDNA using M-MLV Reverse Transcriptase (Life Technologies) and oligo-dT primers (Integrated DNA Technologies, Toronto, ON, Canada). Primer and probe sets for target genes (Table 1) were designed using the Primer Express software (Life Technologies) and expression was analyzed on a StepOne system (Life Technologies) over 40 amplification cycles (15 s for 95°C, 45 s for 60°C). The equivalent of 20 ng of RNA was used in each reaction and performed as duplex reactions; target and GAPDH internal control amplified in the same sample. Data was obtained from three independent experiments and expressed as relative to GAPDH expression.

**Table 1:** Primers and probes used for qPCR.

Target Gene	Forward Primer	Reverse Primer	Probe
GAPDH	5′- TCG TGG AAG GAC TCA TGA C −3'	5'- CCA TCA CGC CAC AGT TT −3'	5'-/5MAXN/AGT CCA TGC CAT CAC TGC CAC/3IABlk_FQ/-3'
C-KIT	5'- CAG ATT TCA GAG AGC ACC AAT CA -3'	5'- AAT GGT CTA CCA CGG GCT TCT -3'	5'-/56-FAM/TTA CTC CAA CTT AGC AAA CTG CAG CCC CAA/36-TAMSp/-3'

### Flow cytometric analysis

For analysis of KIT expression, HMC-1.1, HMC-1.2 and LAD2 cells were treated with NaBu (0–1000 µM) for 3 days at 37°C and 5% CO_2_, then washed in 0.1% BSA-PBS, resuspended at 2  ×  10^5^ cells/ml in the same buffer, and subsequently incubated for 1 h with either PE-conjugated anti-CD117 antibody (eBioscience, San Diego, CA) or mouse IgG isotype control (eBioscience, San Diego, CA) antibody at 4°C in the dark. Cells were washed twice then resuspended in 0.1% BSA-PBS and measured on a FACSArray Flow Cytometer, and data analysis was performed using FlowJo software. Results are reported as Mean Fluorescent Intensity (MFI).

For LIVE/DEAD analysis and tryptase content, untreated cells or those treated with NaBu as indicated in the figure legends were prepared for flow cytometry using round-bottom 96-well plates (Sarstedt, Montréal, QC, Canada). To measure cell viability, cells were resuspended in 100 µl PBS and stained with 5 µl of 1:40 diluted LIVE/DEAD Fixable Near-IR Dead Cell Stain Kit (Molecular Probes, Waltham, MA United States) for 30 min at 4°C. All cells were fixed using 10% formalin (Sigma-Aldrich) for at least 20 min at room temperature and were resuspended in 2% FBS + 1 mM ethylenediaminetetraacetic acid (EDTA) in PBS prior to analysis. For cells used for intracellular tryptase detection, fixed cells (stained with or without LIVE/DEAD Fixable Near-IR Dead Cell Stain or anti-human CD117-PE antibody) were blocked using 2% FBS + 1 mM EDTA in PBS for at least 16 h at 4°C followed by permeabilization with 0.1% saponin in PBS for 10 min at 4°C. The permeabilized cells were first incubated with 1.75 µl mouse anti-tryptase antibody (clone: G3; Millipore, Etobicoke, ON, Canada) in 0.1% saponin in PBS for 20 min at 4°C followed by staining with 2 µl Alexa Fluor 488-conjugated F(ab')2-goat anti-mouse IgG secondary antibody (Invitrogen, Waltham, MA, United States) for 20 min at 4°C. The cells were resuspended in 2% FBS + 1 mM EDTA in PBS prior to analysis. Compensation controls were prepared as follows: one drop of ArC (Amine Reactive Compensation Beads; Molecular Probes) and 5 µl 1:40 diluted LIVE/DEAD Fixable Near-IR Dead Cell Stain, one drop of ArC Total Antibody Compensation Beads and 2 µl Alexa Fluor 488-conjugated F(ab')2-goat anti-mouse IgG were incubated at room temperature for 30 min protected from light, washed with 1.5 ml PBS, centrifuged at 250  ×  *g* for 5 min, then resuspended with 250 µl flow buffer to which one drop of the respective negative beads were added. Alternatively, LIVE/DEAD-stained dead cells (positive control) were prepared by initially fixing cells with 10% formalin at 37°C for 15 min followed by staining with 5 µl of 1:40 diluted LIVE/DEAD Fixable Near-IR Dead Cell Stain Kit for 30 min at 4°C and then a final fixation step (10% formalin, 20 min at room temperature). All samples for flow cytometry were analyzed using a LSRFortessa flow cytometer equipped with 405 nm, 488 nm, and 633 nm lasers and a High Throughput Sampler (BD Biosciences) using the following voltages: FSC: 210, SSC: 240, AlexaFluor488: 320, PE: 400, and APC-Cy7: 430. All data analysis was performed using FlowJo v10.2 software (FlowJo LLC, Ashland, OR, United States). The gating procedure involved debris exclusion using SSC-A vs. FSC-A followed by doublet discrimination using FSC-H vs. FSC-A. The percentage of LIVE/DEADLo cells in the single cell populations were used to measure viability. The average number of events analyzed for cell viability were: 0–0.25 mM, 17,196; 0.5 mM, 17,288; 1.0 mM, 16,034; 2.0 mM, 9,953 and the average number of events analyzed for intracellular tryptase expression were: 0–0.1 mM, 45,456; 0.5 mM, 45,654; 1.0 mM, 42,888.

### Histamine assay

HMC-1.1 and HMC-1.2 cells were either left untreated or were treated with the indicated concentrations of NaBu, trichostatin A (TSA; Cayman Chemical, Ann Arbor, MI, United States), suberoylanilide hydroxamic acid (SAHA; Cayman Chemical), or DMSO (0.0004% final) for four days. The cells were collected in the culture media, pelleted at 200  ×  *g* for 5 min, washed with HEPES buffer (10 mM HEPES, 137 mM NaCl, 2.7 mM KCl, 5.6 mM glucose, 5.6 mM Na_2_HPO_4_, 1.8 mM CaCl_2_, and 1.3 mM MgSO_4_ at a pH of 7.4), and pelleted again at 200  ×  *g* for 5 min. The cells were resuspended in HEPES buffer to a concentration of 1  ×  10^6^ cells/ml and 0.4–1  ×  10^5^ cells were lysed using 0.05% Triton X-100 for 20 min on ice. In a black microplate, 30 µl cell lysate and 30 µl HEPES buffer or 60 µl of histamine dihydrochloride standards in HEPES buffer (7.8–500 ng/ml; Sigma-Aldrich) were added to duplicate wells, combined with 12 µl 1M sodium hydroxide and 2 µl 10 mg/ml *o*-phthalaldehyde (Sigma-Aldrich) in methanol, and incubated for 4 min at room temperature. Subsequently, 6 µl 3M hydrochloric acid was used to stop the reaction and the fluorescence intensity was measured using 360 nm (excitation) and 450 nm (emission) settings with a BioTek Synergy H1M plate reader (Winooski, VT, United States). Lower limit of detection for this assay is approximately 5–7 ng/ml (34, 35).

### Transmission electron microscopy

Untreated or NaBu-treated cells were fixed overnight at 4°C using 2% paraformaldehyde +2.5% glutaraldehyde in PBS, pH 7.4 (EM fixation buffer). After repeated washes, the cells were treated with 1% osmium tetroxide in PBS at room temperature for one hour. The cells were then sequentially dehydrated at room temperature with ethanol (30%, 50%, 70%, 90%, and 100%), 10 min per step. The cells were infiltrated with pure LR white in 100% ethanol (1:1 ratio) overnight at room temperature, followed by infiltrated with fresh LR white for one hour, and then embedded with fresh LR white and polymerized at 55°C for 24 h. The cells embedded in resin were sectioned using a microtome generating ultra-thin sections (∼100 nm) on bared copper TEM grids. Thin sections were coated with 3 nm of carbon using a Precision Etching and Coating System (model 682; Gatan, Pleasanton, CA, United States). All bright field TEM images were obtained at 100 kV in H7700 TEM (Hitachi High-Technologies, Tokyo, Japan).

### Statistical analysis

Experiments were conducted at least three independent times, using three independent cultures of cells and three independent stocks of chemicals. All values are presented as mean ± standard error of the mean (SEM). Data were analyzed using Student's *t*-test. Differences were considered statistically significant as follows: not significant, *p* > 0.05; *0.05 ≥ *p* > 0.01; **0.01 ≥ *p* > 0.001; ***p ≥ 0.001.

## Results

### Sodium butyrate inhibits HMC-1.1 and HMC-1.2 cell proliferation, viability, and metabolic activity

**Figure 1:**
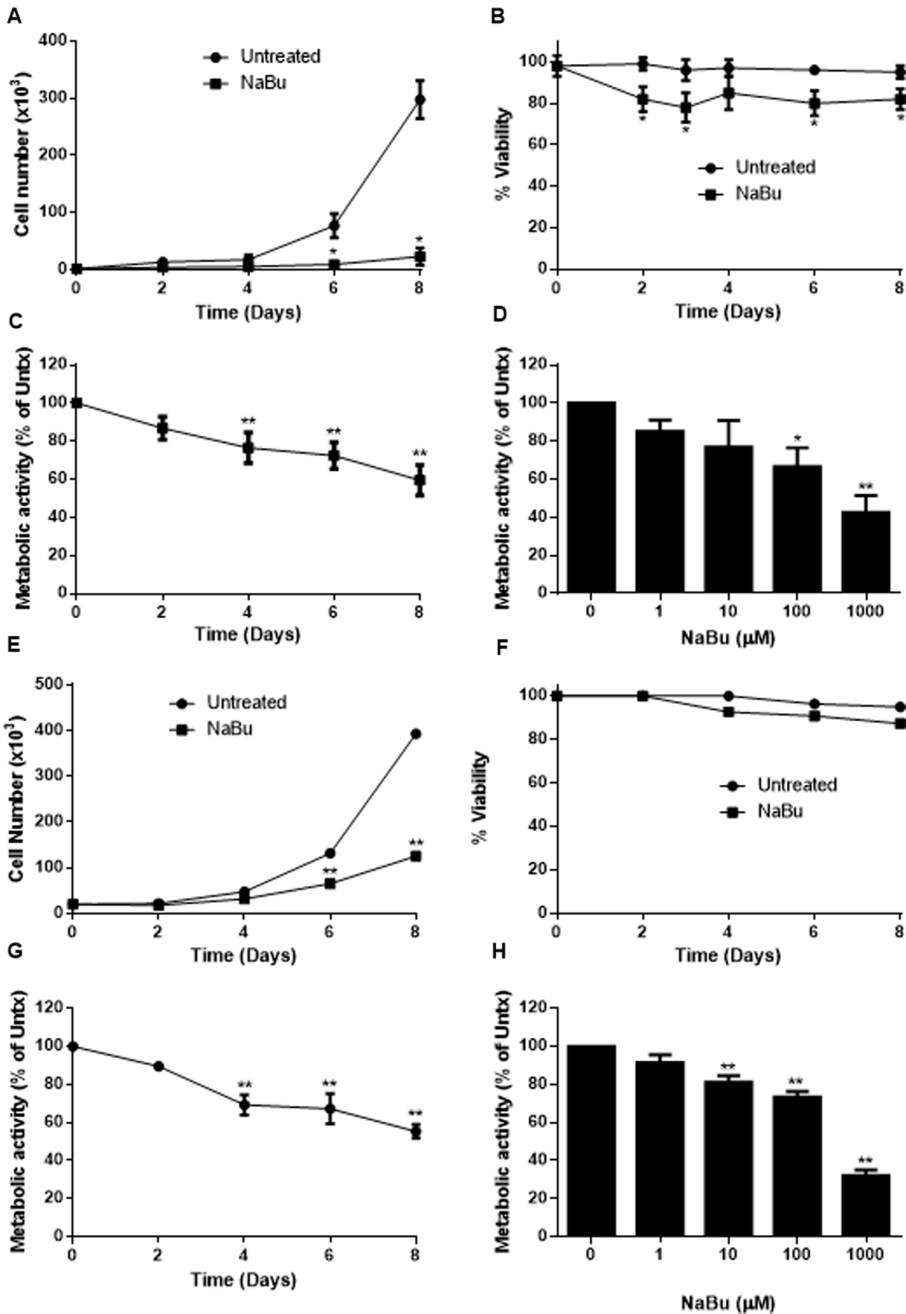
Sodium butyrate (NaBu) inhibits HMC-1.1 and HMC-1.2 cell proliferation and metabolic activity. HMC-1.1 cells were cultured with NaBu (100 µM) for an 8-day time course, and cell number (**A**) and cell viability (**B**) were measured (*n* = 3). HMC-1 cells were cultured with NaBu (100 µM) for up to 8 days, and a time course metabolic activity (**C**) and (**D**) a NaBu (0–1000 µM) dose-response experiment was performed using XTT assay (*n* = 5). HMC-1.2 cells were cultured with NaBu (100 µM) for an 8-day time course, and cell number (**E**) and cell viability (**F**) were measured (*n* = 3). (**G**) Cells were cultured with NaBu (100 µM) for 8 days, and a time course metabolic activity (*n* = 5) and (**H**) a NaBu (0–1000 µM) concentration course was measured using XTT assay (*n* = 5). *p < 0.05, **p < 0.01.

Human mast cell lines HMC-1.1 and HMC-1.2 were cultured with 100 µM NaBu and cell proliferation was measured using a standard trypan blue cell counting protocol (Figures 1A–E). NaBu significantly inhibited HMC-1.1 proliferation after six days in culture and by eight days, very few cells were present (less than 10% of those in the untreated group; Figure 1A). NaBu decreased cell viability by approximately 20% and this effect remained consistent for up to eight days (Figure 1B). An examination of cellular metabolic activity showed that NaBu decreased overall metabolic activity of HMC-1.1 after 4 days (Figure 1C) and in a concentration-dependant manner (Figure 1D). NaBu decreased HMC-1.1 metabolic activity by approximately 25% by day four and up to 40% by day eight (Figure 1C). Furthermore, this decrease in metabolic activity was dependent on NaBu concentration and had a calculated IC_50_ of approximately 602 µM (Figure 1D).

NaBu inhibited cell proliferation and viability of HMC-1.1 cells more potently than HMC-1.2 cells. NaBu inhibited proliferation of HMC-1.2 cells by 50% by day six (Figure 1E) and 70% by day eight, but viability of the HMC-1.2 cells decreased by only 5% by day eight (Figure 1F). Again, metabolic analysis showed that NaBu (100 µM) decreased metabolic activity by 40% by day eight (Figure 1G), and this effect was concentration-dependent (Figure 1H). Therefore, NaBu had similar effects on metabolic activity in both HMC-1.1 and HMC-1.2 cells.

### Sodium butyrate arrests the cell cycle of HMC-1.1 and HMC-1.2 cells

**Figure 2:**
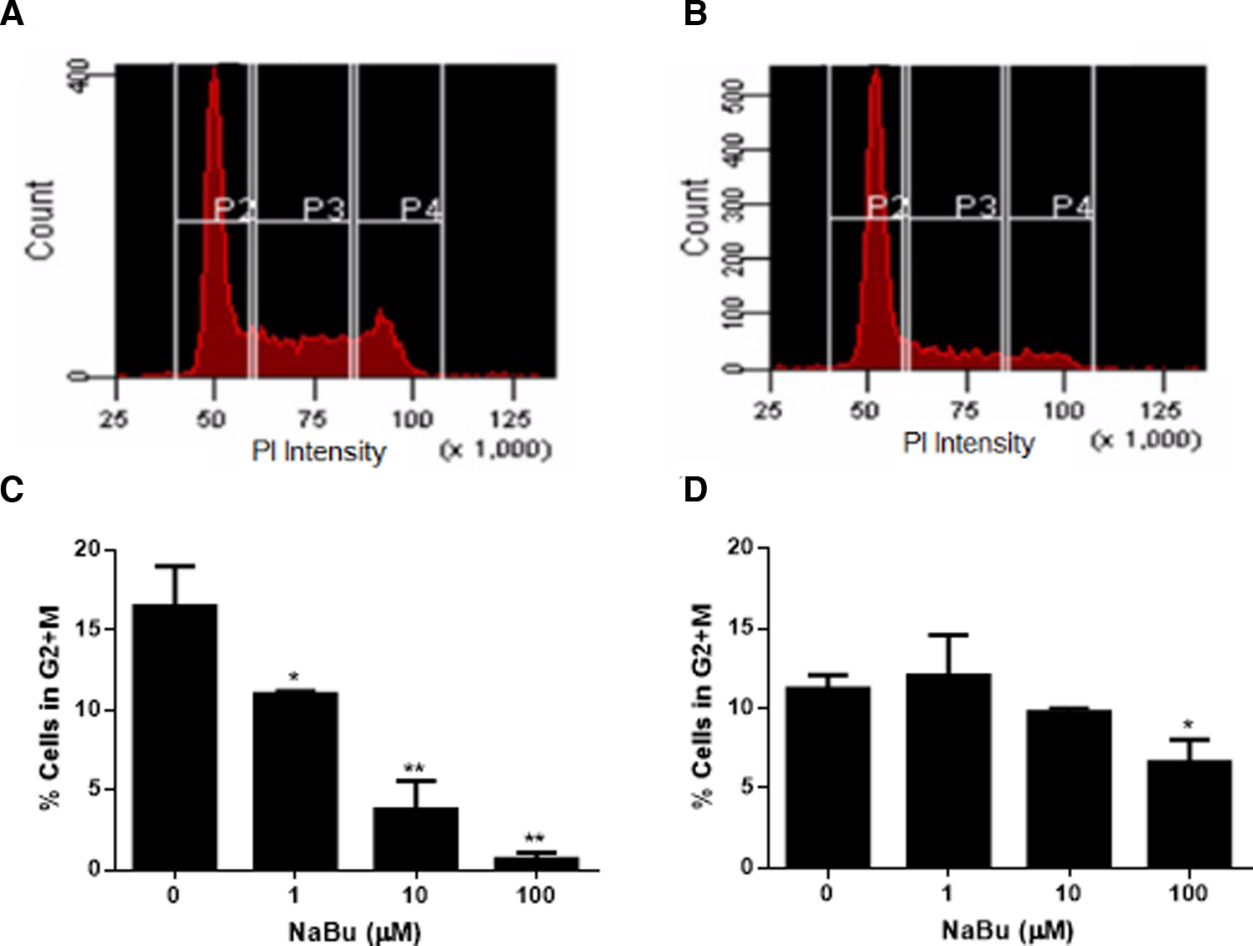
Sodium butyrate (NaBu) blocks the cell cycle in HMC-1.1 and HMC-1.2 cells. The cell cycle of untreated (**A**) and NaBu-treated (100 µM; **B**) HMC-1.1 cells were analyzed by flow cytometry for cell cycle progression. In this representative histogram, gate P2 represents cells in the G_0_ or G_1_ phase, P3 represents cells doubling their DNA content in the S phase and P4 represents cells in the G2 and M phases. HMC-1.1 (**C**) and HMC-1.2 (**D**) cells were cultured in NaBu (0–100 µM) for 24 h and the cell cycle was analyzed by propidium iodide (PI) staining of RNA and flow cytometry (*n* = 3). *p < 0.05, **p < 0.01.

NaBu is sometimes used for cell cycle synchronization (36, 37) because it regulates a number of genes related to the cell cycle and cell division such as H3 histone, C-Ha-ras, ornithine decarboxylase (36), cyclins and the Fas pathway (38). Cell division requires duplication of DNA, and this double diploid state can be quantified by incubating fixed and permeabilized cells with the stoichiometric dye, propidium iodide. Using this approach to measure cell cycle progression of NaBu-treated cells, our data showed that NaBu treatment reduced the number of HMC-1.1 cells that had progressed into the G2 and M phases of the cell cycle, thus reducing the number of cells undergoing cell division (Figures 2A,B). Furthermore, this effect increased with the concentration of NaBu such that 10 µM of NaBu decreased the number of HMC-1.1 cells in the G2 and M phases by 50% and 100 µM almost completely blocked progression of the cells into the G2/M phase (Figure 2C).

NaBu also inhibited the cell cycle progression of HMC-1.2 but this effect was not as pronounced as the effect observed with HMC-1.1 cells. As shown in Figure 2D, only the highest concentration tested of NaBu (100 µM) led to a significant decrease in the percentage of cells in the G2/M, which corresponded to approximately 50% fewer cells continuing through the cell cycle (Figure 2D).

### Sodium butyrate reduces KIT (CD117) expression by HMC-1.1 and HMC-1.2 cells

**Figure 3:**
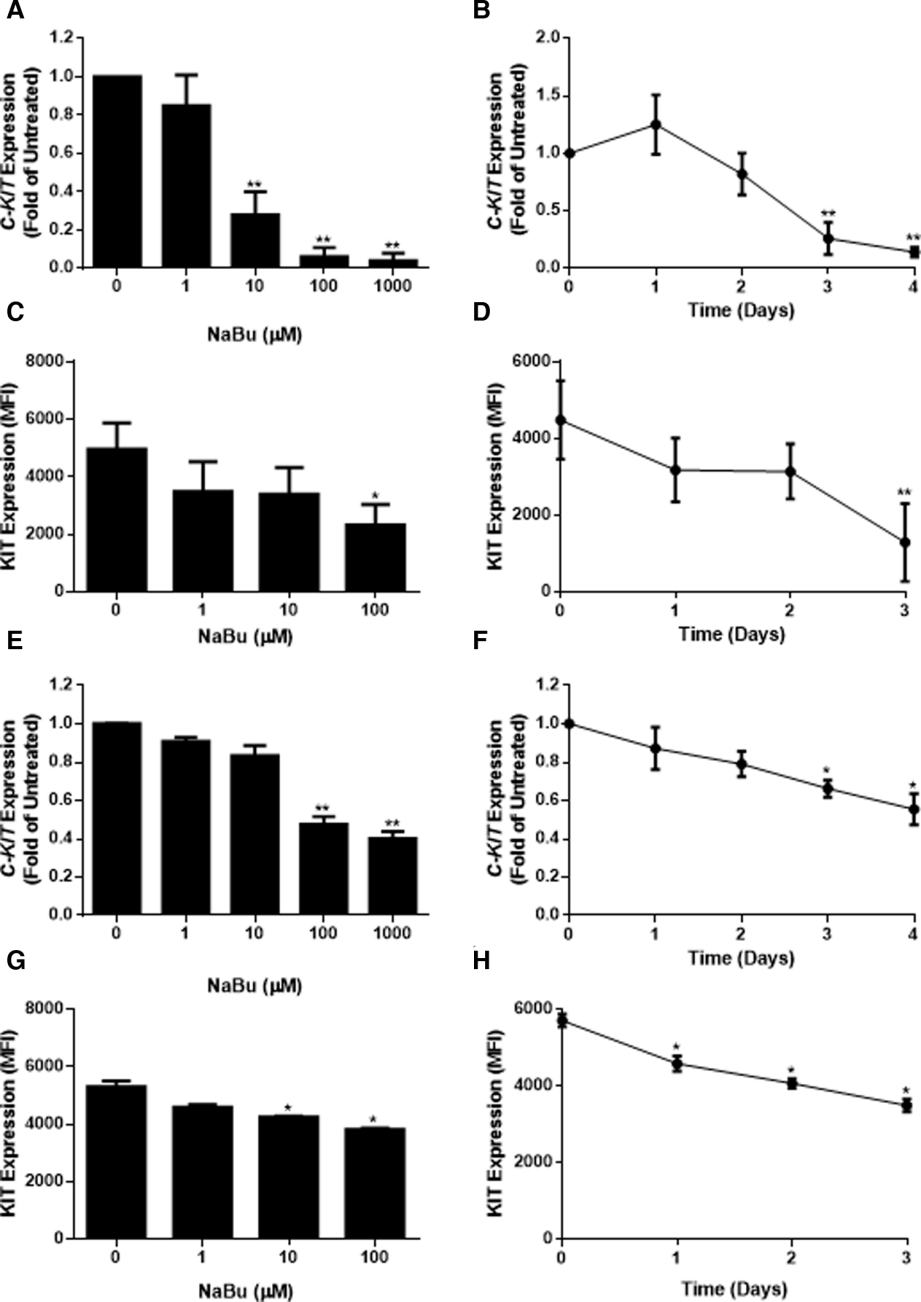
Sodium butyrate (NaBu) inhibits *KIT* (gene) and KIT (protein) expression in HMC-1.1 and HMC-1.2 cells. In HMC-1.1 cells, *C-KIT* expression was measured using qPCR following culture in (**A**) NaBu (0–1000 µM) for three days or (**B**) NaBu (100 µM) for up to four days (*n* = 3). KIT protein expression was measured by flow cytometry in HMC-1 cells cultured with (**C**) NaBu (0–100 µM) for three days or (**D**) 100 µM of NaBu for up to three days (*n* = 3). HMC-1.2 cells were treated with NaBu (0–1000 µM) for three days (**E**) or NaBu (100 µM) for up to four days (**F**), and *C-KIT* expression was measured using qPCR (*n* = 3). KIT protein expression was measured by flow cytometry following treatment with (**G**) NaBu (0–100 µM) for three days or (**H**) NaBu (100 µM) for up to three days (*n* = 3). *p < 0.05, **p < 0.01. MFI = Mean Fluorescent Intensity.

Due to its ability to regulate the expression of several growth receptors (39, 40) and differentiation factors (41), NaBu is sometimes used to differentiate transformed cell lines into more mature phenotypes. We hypothesized that NaBu might inhibit the proliferation and division of HMC-1 cells by modulating the expression of *C-KIT*; which encodes a critical receptor tyrosine kinase in mast cell growth, differentiation, and function (25). Treatment with 100 µM NaBu inhibited expression of *C-KIT* mRNA in HMC-1.1 cells by greater than 70% (Figure 3A) and this effect required at least three days of culture with NaBu (Figure 3B).

Since changes in mRNA expression do not always correlate with changes in protein expression (42), particularly in HMC-1 cell lines (43), flow cytometry was used to measure the surface expression of the KIT receptor during NaBu treatment. NaBu (0–100 µM) treatment decreased the expression of KIT protein (Figure 3C) on the surface of HMC-1.1 cells by 50%, and this effect was observed after three days of treatment (Figure 3D).

Similar to its effect on HMC-1.1 cells, NaBu downregulated the expression of *C-KIT* mRNA by HMC-1.2 cells in a concentration- and time-dependent manner (Figures 3E,F). NaBu inhibited the expression of the KIT receptor on the surface of HMC-1.2 cells (Figure 3G) by 30%, and this occurred after three days of treatment (Figure 3H). However, compared to HMC-1.1, the effect of NaBu on HMC-1.2 KIT expression was less pronounced, requiring higher concentrations and longer treatment times for similar levels of inhibition.

### Sodium butyrate inhibits LAD2 proliferation and metabolic activity

LAD2 is a mast cell line that is considered to have a more mature phenotype than HMC-1 cells since LAD2 cells have a functional Fc*ε*R1 receptor and the ability to degranulate in response to cross-linked IgE (28). LAD2 do not harbour the V560G or D816V mutations in their KIT receptor and therefore require SCF for survival and proliferation. We next determined whether NaBu had effects on LAD2 cell proliferation and viability.

**Figure 4:**
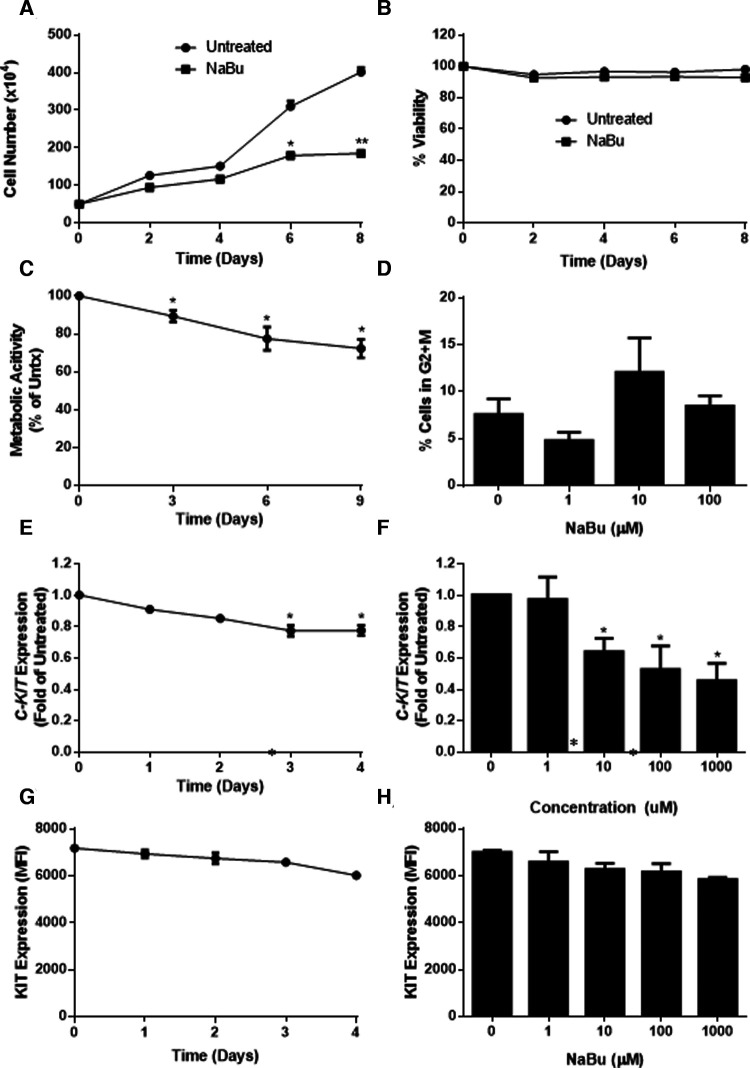
Sodium butyrate (NaBu) inhibits proliferation, metabolic activity, and cell cycle in LAD2, as well as *C-KIT* mRNA and KIT protein expression. LAD2 cells were cultured in NaBu (100 µM) for eight days, during which (**A**) total cell number (*n* = 3) and (**B**) cell viability were measured (*n* = 3). (**C**) The effect of NaBu on metabolic activity was measured using XTT assay (100 µM over nine days; *n* = 5). (**D**) Cells were cultured in NaBu (0–100 µM) for 24 h, and the cell cycle was analyzed by propidium iodide staining and flow cytometry (*n* = 3). (**E**) Cells were cultured in NaBu (0–1000 µM) for three days and *c-Kit* expression was measured using qPCR (*n* = 3). (**F**) Cells were cultured in NaBu (100 µM) for up to four days. and *C-KIT* expression was measured by qPCR (*n* = 3). (**G**) Cells were cultured with NaBu (100 µM), and KIT protein expression was measured using flow cytometry (*n* = 3). (**H**) Cells were cultured with NaBu for three days, and KIT protein expression was measured using flow cytometry (*n* = 3). *p < 0.05, **p < 0.01.

Similar to HMC-1 cells, NaBu inhibited the proliferation of LAD2 cells by as much as 50% by day eight of treatment (Figure 4A). However, NaBu had little effect on LAD2 cell viability, and 92% of the NaBu-treated cells remained viable on day 4 (Figure 4B). An analysis of metabolic rate showed that NaBu decreased the metabolic activity of LAD2 cells by almost 25% by day six of treatment (Figure 4C), similar to the effect of NaBu on HMC-1.1 and HMC-1.2 cells observed in Figure 1.

Since NaBu arrested the cell cycle in HMC-1 cells, we next determined if NaBu had the same effect on the cell cycle in LAD2 cells. Interestingly, NaBu had no significant effect on LAD2 cell cycle progression to the G2/M phase. However, in both the NaBu-treated and untreated groups (Figure 4D), the majority (more than 70%) of the cells remained in the G1/G0 phase of the cell cycle, perhaps reflecting the slow proliferation and long doubling time (approximately 10 days) of the LAD cell line (28).

### Sodium butyrate downregulates expression of KIT by LAD2

Since NaBu had significant effects on *C-KIT* expression by HMC-1.1 and HMC1.2 cells, we next determined the effect of NaBu on *C-KIT* expression in LAD2 cells. NaBu inhibited the expression of *C-KIT* mRNA in LAD2 cells in both a concentration- and time-dependent manner. NaBu treatment decreased the expression of *C-KIT* compared to untreated (Figure 4E), and by three days of treatment *C-KIT* expression decreased by approximately 50% (Figure 4F).

NaBu also decreased the surface expression of KIT protein on LAD2 cells in both a concentration- and time-dependent manner. NaBu treatment reduced the expression of KIT on the surface of LAD2 cells by 10% (10 µM NaBu) and 17% (1000 µM NaBu; Figure 4G) compared to untreated cells. The inhibitory effect of NaBu on KIT surface expression increased with treatment time and reduced KIT expression by 17% by day 4 (Figure 4H).

### Sodium butyrate moderately increases tryptase and histamine in HMC-1.2

Since NaBu decreased the proliferation and metabolic activity of HMC-1 cells, we next determined whether NaBu could modify some of the hallmarks of mast cell differentiation, such as tryptase and histamine expression. NaBu has been used to differentiate HL-60 into eosinophil-like cells, but usually at quite high concentrations of 500 µM or higher (44). Therefore, for the next set of experiments, HMC-1.1 and HMC-1.2 were treated with higher concentrations of NaBu (100 µM, 500 µM and 1.0 mM) to determine whether higher concentrations would have more potent effects on HMC-1 differentiation. Although 500 µM NaBu had no significant effect on HMC-1.1 viability, it significantly decreased HMC-1.2 cell viability by day eight (Supplementary Figures S1A,B). Similarly, flow cytometric analysis of cell viability indicated that 500 µM and 1.0 µM decreased viability of HMC-1.2 cells by day eight.

**Figure 5:**
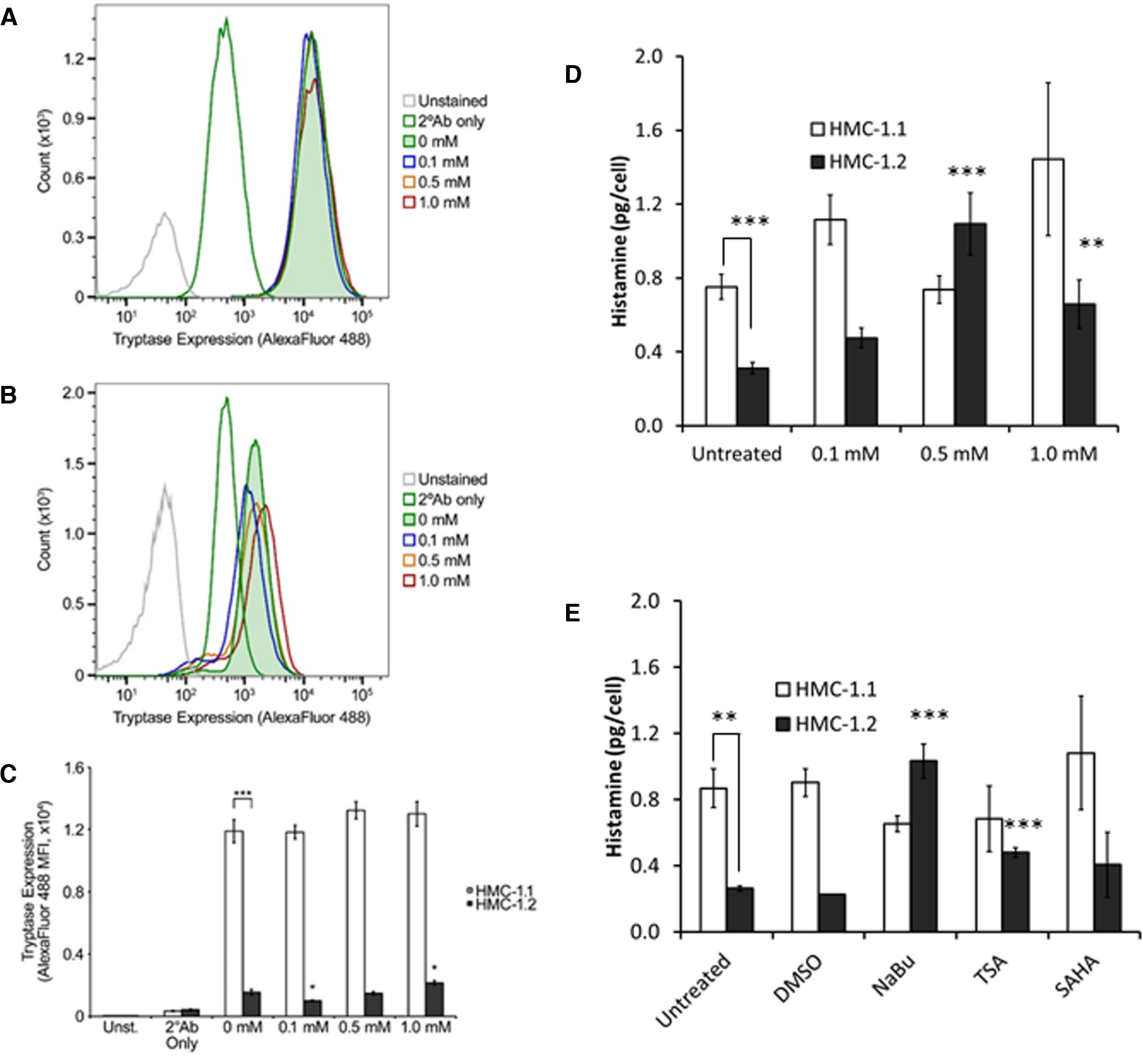
Tryptase and histamine content of HMC-1.1 and HMC-1.2 cells treated with sodium butyrate (NaBu) and HDACi. Representative flow cytometry data from HMC-1.1 (**A**) or HMC-1.2 (**B**) cells untreated or treated with the indicated concentrations of NaBu for four days and stained with Alexa Fluor 488-conjugated secondary antibodies (2°Ab) ± anti-tryptase antibodies are shown. Number of events shown for HMC-1.1 in panel A: Unstained, 14,975; 2°Ab only, 46,482; 0 mM, 42,150; 0.1 mM, 41,745; 0.5 mM, 41,547; 1.0 mM, 39,822. Number of events shown for HMC-1.2 in panel B: Unstained, 47,209; 2°Ab only, 47,009; 0 mM, 45,657; 0.1 mM, 44,131; 0.5 mM, 43,093; 1.0 mM, 41,409. (**C**) Summary of the tryptase/Alexa Fluor 488 median fluorescence intensity (MFI) values obtained from untreated and NaBu-treated HMC-1.1 and HMC-1.2 cells (*n* = 5). (**D**) HMC-1.1 and HMC-1.2 cells were treated with the indicated concentrations of NaBu for four days and were assessed for histamine levels *n* = 7, except for 1 mM treatment where *n* = 3. (**E**) HMC-1.1 and HMC-1.2 cells were left untreated or treated with DMSO (vehicle) control, 0.5 mM NaBu, 100 nM trichostatin A (TSA), or 200 nM suberoylanilide hydroxamic acid (SAHA) for four days and were assessed for histamine levels **p < 0.01; ***p < 0.005.

A preliminary analysis of tryptase content in HMC-1 sublines indicated that HMC-1.1 contain much higher amounts of tryptase than HMC-1.2 (Figures 5A,B). NaBu did not increase tryptase in HMC-1.1 cells (Figure 5A). However, NaBu treatment moderately increased tryptase content of HM-1.2 cells (Figures 5B, 5C) when they were treated with 1.0 mM NaBu.

Similar to the tryptase data, HMC-1.1 contained approximately two times more histamine per cell than HMC-1.2 (Figure 5D). NaBu treatment increased histamine content of HMC-1.2 cells in a concentration-dependent manner, reaching maximum induction at 500 µM NaBu (Figure 5D). NaBu did not increase histamine content of HMC-1.1, and although there was a trend of increased histamine content of HMC-1.1 cells treated with 1.0 mM NaBu, this was not significant when compared to the untreated cells. Lastly, two other HDACi, trichostatin A (TSA) and suberoylanilide hydroxamic acid (SAHA), were tested for their ability to modify histamine content in HMC-1 cells (Figure 5E). TSA increased histamine in HMC-1.2 cells but had no effect on HMC-1.1. SAHA had no significant effect on either HMC-1.1 or HMC-1.2 compared to untreated cells.

### Sodium butyrate alters the ultrastructure of granules in the cytoplasm

**Figure 6:**
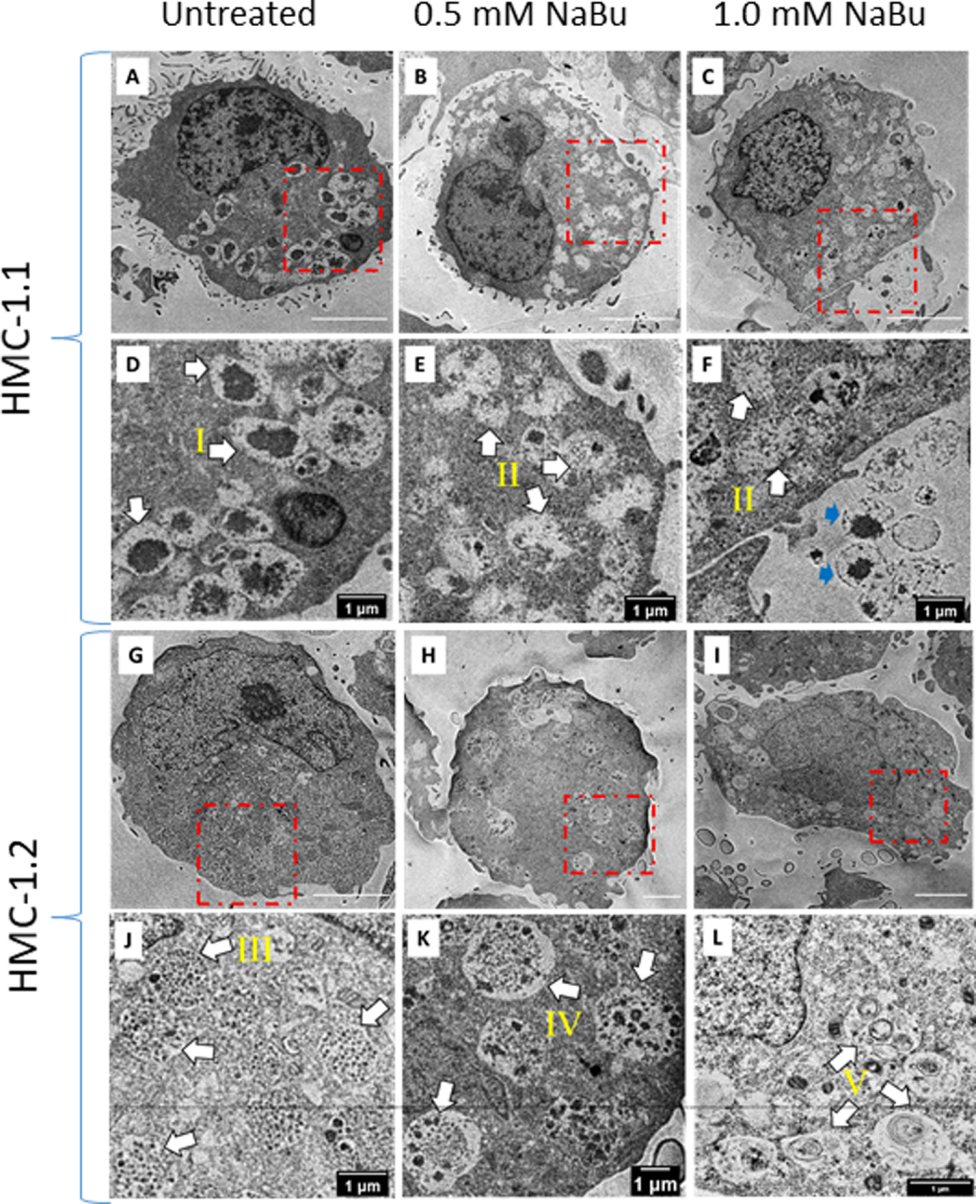
Sodium butyrate (NaBu) treatment of HMC-1.1 and HMC-1.2 cells causes changes in granule phenotype. HMC-1.1 (**A–F**) or HMC-1.2 (**G–L**) cells were untreated (**A** and **G**) or treated with 0.5 mM (B and H) or 1 mM (C and I) NaBu for four days followed by fixation, sectioning, and imaging by transmission electron microscopy. (**D–F**) and (**J–L**) are corresponding enlarged images from the areas marked in red squares in (**A–C**) and (**G–I**). Five types of morphologically distinct granules in the cytoplasm are identified by white arrows: electron-dense core surrounding by sparse particulates (type I), less electron-dense and more electron-lucent (type II), uniform lumen/particulates (type III), mixture of electron-dense vesicles and particulates(type IV) and scroll-like or multi-lamellar vesicles (type V). Extracellular vesicles with electron-dense core are identified by blue arrows in F.

Since NaBu caused an increase of histamine and tryptase content, we determined whether this was associated with ultrastructural changes with secretory granules in the cytoplasm. Figure 6 shows the intracellular ultrastructure of HMC-1.1 and HMC-1.2 untreated or treated with NaBu. We identified and classified granules into five types according to their ultrastructure (I, II, III, IV and V). The majority of granules in the cytoplasm of untreated HMC-1.1 have electron-dense core surrounded by sparse particulates (type I, Figures 6A–D). With NaBu treatment the electron-dense core vanished and the granules became more electron-lucent (type II, Figures 6 B&E, C&F). Interestingly, extracellular vesicles with electron-dense core were observed after 1.0 mM NaBu treatment (blue arrows in Figure 6F). Whereas, granules in untreated HMC-1.2 are uniformly filled with 60–80 nm particulates (type III, Figures 6G–J), which making the membrane of granules is hardly to tell apart from cytosol at lower magnification. After treatment with 0.5 mM NaBu, large size particulates (100–250 nm) increased but still sparsely distributed within compartments (type IV, Figures 6H–K) and some vesicles appeared in the granules. Multivesicular body (MVB) were also observed (black arrow, Figure 6H). While with 1.0 mM NaBu treatment, scroll-like or multilamellar vesicles (MLV) are shown in the majority of granules (type V, Figures 6I–L).

## Discussion

Our data indicates that NaBu differentially inhibited the proliferation of three human transformed mast cell lines in both a time- and concentration-dependent manner and which was associated with changes in tryptase and histamine content (Table 2). Furthermore, our data shows that this effect was associated with cell cycle arrest and a decrease in proliferation, without a significant change in cell viability. HDACi such as NaBu cause both induction and repression of gene expression (45) at an epigenetic level, by increasing acetylation of histones and/or non-histone HDAC substrates that include a number of transcription factors and other important proteins. Butyrate is the smallest known HDACi and contains a simple three carbon “spacer” attached to a carboxylic acid group which enters the active site of HDAC and forms a bidentate ligand with the buried zinc atom (46). Butyrate can inhibit HDAC activity at high micromolar to low millimolar concentrations *in vitro*, and (47–51) such concentrations of butyrate are possible in the gastrointestinal tract, where butyrate serves as the principal oxidative fuel for colonocytes (47–51). Mast cells are present in the gastro-intestinal tract in their capacity as components of the immunological surveillance system. Mastocytosis, a clonal proliferation of mast cells, results from a mutation in the KIT pathway and presents with cutaneous symptoms, and in some cases, patients present with gastro-intestinal symptoms (52). Interestingly gastro-intestinal stromal tumours (GIST), a rare tumor located in the gastro-intestinal tract, are often associated with a mutation in the KIT receptor similar to those identified in our mast cell lines (53).

**Table 2:** Summary of NaBu effects on human mast cell lines.

	HMC-1.1	HMC-1.2	LAD2
Proliferation (Day 8)	92.3 ± 5%^1^	67.9 ± 1.2%^1^	46.1 ± 1.1%^1^
Viability (Day 8)	87.0 ± 5%^2^	92.4 + 0.5%^2^	94.7 ± 0.9%^2^
Metabolic Activity (Day 8)	59.6 ± 8%^1^	55.3 ± 3.5%^1^	72.3 ± 4.8%^1^
*C-KIT* Expression (1000 µM)	0.04 ± 0.04^3^	0.40 ± 0.04^3^	0.45 ± 10.9^3^
*C-KIT* Expression (Day 4)	0.14 ± 0.04^4^	0.55 ± 0.08^4^	0.77 ± 0.03^4^
KIT Expression (100 µM)	47.1 ± 4.4%^5^	71.9 ± 1.8%^5^	88.1 ± 5.1%^5^
KIT Expression (Day 3)	28.7 ± 22.6%^6^	61.2 ± 2.9%^6^	91.6 ± 1.8%^6^
Inhibition of cells in G2/M (100 µM)	95.7 ± 2.4%^7^	58.9 ± 12.5%^7^	N/A

^1^ Compared to untreated cells, which were considered 100%.

^2^ Percent of cells that were trypan blue negative.

^3^ Ratio of C-KIT expression of cells treated with 1000 µM NaBu for 3 days, compared to untreated cells.

^4^ Ratio of C-KIT expression of cells treated with 100 µM NaBu for 4 days, compared to untreated cells.

^5^ Ratio of C-KIT expression of cells treated with 100 µM NaBu for 3 days, compared to untreated cells.

^6^ Ratio of C-KIT expression of cells treated with 100 µM NaBu for 3 days, compared to untreated cells.

^7^ Percent reduction in the number of cells in G2/M, compared to untreated cells.

In our study, NaBu inhibited the proliferation of three transformed mast cell lines: HMC-1.1, HMC-1.2 and LAD2. Each of these cell lines has different mutations in KIT, suggesting that these different effects of NaBu may be related to the function of KIT. Carson et al*.* reported that when a subset of natural killer (NK) cells that constitutively express *C-KIT* are treated with SCF they upregulate bcl-2 which in turn suppresses apoptosis (54), showing that activation of KIT results in the upregulation of bcl-2 and prevention of apoptosis. It is therefore possible that NaBu may modify the function of these apoptotic pathways *via* the KIT receptor. Multiple transformed cell lines are susceptible to NaBu-mediated cell death through various mechanisms including the bcl-2 and Ras pathways (38, 55).

The different effects of NaBu on these cell lines may also be explained by their substantially different growth rates. Nilsson et al*.* found that HMC-1 cultured in standard conditions proliferate rapidly (19) and HMC-1.1 increase 50-fold and HMC-1.2 increase 10-fold over seven days. However, LAD2 proliferate very slowly and, when grown in serum-free media supplemented with 100 ng/mL of SCF, they double approximately every two weeks (28). NaBu inhibited the proliferation of all of these cell lines but HMC-1.1 were the most sensitive to NaBu effects, whereas LAD2 were the least sensitive (compare Figures 1A–E, 4A). This supports our hypothesis that NaBu functions by inhibiting the cell cycle of these cells, and thus cells that proliferate less rapidly through the cell cycle are less sensitive to NaBu effects. The inhibitory effect on proliferation in all cases was not related to slight decreases in viability which were similar among the three cell lines (Figures 1B–F, 4B). Metabolic activity was inhibited in all three cell lines in both in a time- and concentration-dependent manner. Metabolic inhibition in HMC-1.1 and HMC-1.2 was greater than LAD2 which correlated to decreased proliferation (Figures 1–G, 4C).

NaBu is a known cell cycle inhibitor, specifically in transformed cell lines (56) and NaBu arrested the cell cycle of HMC-1.1, and to a lesser extent HMC-1.2 (Figure 2). NaBu most profoundly arrested the cell cycle of HMC-1.1 and blocked the progression of HMC-1.2 into the G2 phase by approximately 50%. We were unable to measure the effect of NaBu on LAD2 cell cycle progression, mainly because their extremely slow doubling-time made it difficult to detect any cells in G2 + M (data not shown). However, we speculate that a longer treatment time of up to two weeks may be more informative.

The KIT receptor for the ligand SCF is the main growth factor for mast cells and KIT mutations can result in deregulation, auto-activation, and subsequently proliferation which is independent of SCF. HMC-1.1 and HMC-1.2 were identified by Sundstrom et al*.* who showed that these cells have different mutations in the *C-KIT* gene (57). *C-KIT* is a recognized proto-oncogene that has been identified in GIST, acute myelogenous leukemia (AML) and mastocytosis (58) and the KIT protein is the target of clinical tyrosine kinase inhibitors such as imatinib. Mutations in the Kit receptor were classified by Longley et al*.* as being of two types, regulatory type (ex. V560G and enzyme pocket type (ex. D816V) (59), whose classification can be used to direct treatment. Mastocytosis is associated with several mutations, including the D816V mutation (60). Ma et al*.* found that imatinib selectively inhibits the growth of HMC-1.1 (V560G) compared to HMC-1.2 (V560G, D816V), and suggested that D816V mutation may be resistant to tyrosine kinase inhibitors (61) due to the activating mutation of Asp to Val at codon 816 and subsequent autophosphorylation of KIT. NaBu inhibited KIT expression in all three human mast cell lines, regardless of their mutations (57), suggesting that NaBu may function as a broad-spectrum inhibitor of these mutations.

Our data indicates that NaBu inhibits HMC-1.1 and HMC-1.2 proliferation by causing cell cycle arrest at G2/M. Interestingly, NaBu does not significantly modify cell viability, even after several days of treatment, suggesting that although the cells have stopped proliferating, they are still technically viable and for the most part metabolically active. This decrease in proliferation was associated with a downregulation in *C-KIT* mRNA and KIT protein expression by all three cell types, but with the most significant effect occurring in the rapidly dividing HMC-1 cells. This supports our earlier hypothesis that NaBu, acting as an HDACi, modifies expression of KIT thereby leading to perturbations in the KIT signaling pathway and ultimately loss of proliferation. How these pathways are linked to cell cycle is still an active area of investigation. Furthermore, it is still unknown how NaBu alters KIT expression – whether NaBu causes histone or transcription factor aceylation (62), both known mechanisms of HDACi regulation of gene expression. Several transcription factors or enhancers such as estrogen-induced transcription factor (EGR1), the basic helix-loop-helix transcription factor SCL/Tal1, Runt-related transcription factor 1 (RUNX1) and Far Upstream Binding Protein 1 (FUBP1) are all reasonable candidates and are the subject of future investigation by our team (63–65). Nevertheless, to our knowledge, this is the first demonstration that NaBu decreases *C-KIT* and KIT expression by human mast cells and the first study to show that NaBu alters the cell cycle of transformed human mast cells regardless of their *C-KIT* mutations, leading to a decrease in proliferation.

Recent studies suggest that mutations in genes other than KIT may also contribute to mast cell transformation and this could be linked to changes in methyltransferase activity. Exosome sequencing of a patient with MCL discovered a biallelic loss-of-function mutation in the *SETD2* histone methyltransferase gene which was also present in HMC-1.1 and HMC-1.2 (66). Therefore, HDACi such as NaBu may have multiple targets in HMC-1, targeting multiple genes.

NaBu not only decreases the proliferation of HMC-1.1 and HMC-1.2, it also slightly increases their expression of trypase and histamine content (Figure 5), suggesting that these cells are becoming more differentiated into the mast cell phenotype. The other HDACi that were tested, TSA and SAHA, appeared to have the same effect, although only the TSA increase in histamine content was statistically significant. A recent study by Alanazi et al. has shown that when HMC-1 are induced to undergo apoptosis by HDACi (LLME, staurosporine, UNC-0,638 and UNC-1999), tryptase is released and cleaves histones 3 and 2B, leading to increased proliferation (67). Viability in the Alanazi et al. study was measured using a resazurin-based assay which measures cell metabolic activity similar to the XTT assay used in our analysis. Alanazi et al. observed little change in metabolic activity after 24 h with 100 µM treatment of LLME but observed a significant drop in metabolic activity of HMC-1 treated with the other HDACi, even at concentrations below 1 µM. These results are consistent with our data, showing that HDACi causes a decrease in metabolic activity. Our data also suggest that prolonged treatment of HMC-1 with NaBu causes a slight but measurable increase in tryptase expression. Our analysis did not determine whether this increase was due to increased transcription of the tryptase gene or whether this is due to some changes in localization within the cell, which requires further study. It is possible that the changes in viability are influencing changes in tryptase expression and histamine content. The trypan blue exclusion assay indicates that NaBu causes a drop in viability by approximately 40% for HMC-1.1 and approximately 10%–15% for HMC-1.2 (Supplementary Fig. S1). However, one would predict that tryptase expression and histamine content would decrease in non-viable cells, not increase as our data indicates (Figure 5). Further analysis is required to determine the precise mechanisms of this effect.

In conclusion, our study supports earlier observations and shows that the histone deacetylase inhibitor NaBu attenuated HMC-1 proliferation and metabolic activity. Our study also shows that inhibition of cell proliferation by NaBu was associated with an arrest of the cell cycle and a modest increase in histamine content, tryptase expression, and granularity.

## Data Availability

The original contributions presented in the study are included in the article/**Supplementary Material**, further inquiries can be directed to the corresponding author.
