# Supplementary figures and images for: Sodium butyrate supresses malignant human mast cell proliferation, downregulates expression of KIT and promotes differentiation
Source: Front Allergy. 2023 Mar 10;4:1109717. doi: 10.3389/falgy.2023.1109717 (PMC10036836; doi:10.3389/falgy.2023.1109717)

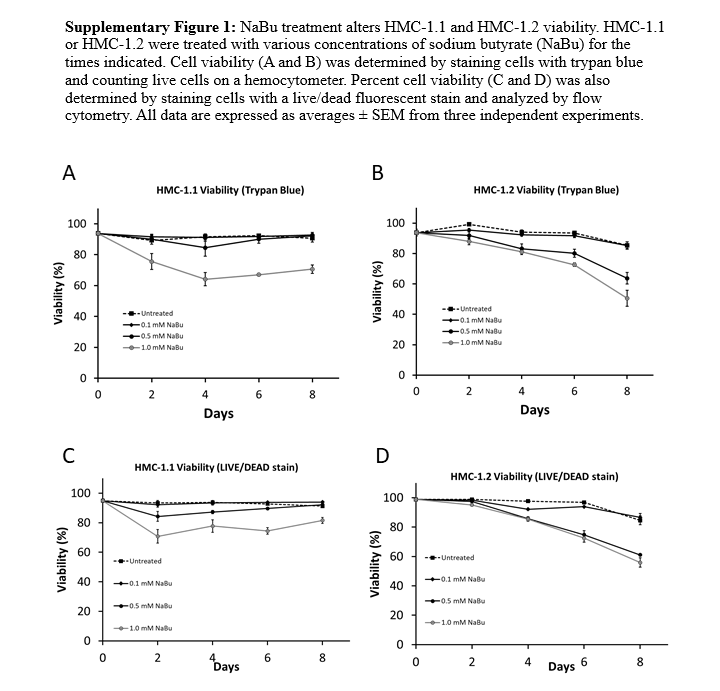

Supplement: Supplementary file 1 [file Image1.tiff]
